# Fear Conditioned Discrimination of Frequency Modulated Sweeps within Species-Specific Calls of Mustached Bats

**DOI:** 10.1371/journal.pone.0010579

**Published:** 2010-05-12

**Authors:** Jie Ma, Robert T. Naumann, Jagmeet S. Kanwal

**Affiliations:** Department of Physiology and Biophysics, Georgetown University, Washington, D. C., United States of America; University of Lethbridge, Canada

## Abstract

Social and echolocation vocalizations of bats contain different patterns of frequency modulations. An adult bat's ability to discriminate between various FM parameters, however, is not well established. Using changes in heart rate (HR) as a quantitative measure of associative learning, we demonstrate that mustached bats (*Pteronotus parnellii*) can be fear conditioned to linear frequency modulated (FM) sweeps typically centered at their acoustic fovea (∼60 kHz). We also show that HR is sensitive to a change in the direction of the conditional frequency modulation keeping all other parameters constant. In addition, a change in either depth or duration co-varied with FM rate is reflected in the change in HR. Finally, HR increases linearly with FM rate incremented by 0.1 kHz/ms from a pure tone to a target rate of 1.0 kHz/ms of the conditional stimulus. Learning is relatively rapid, occurring after a single training session. We also observed that fear conditioning enhances local field potential activity within the basolateral amygdala. Neural response enhancement coinciding with rapid learning and a fine scale cortical representation of FM sweeps shown earlier make FMs prime candidates for discriminating between different call types and possibly communicating socially relevant information within species-specific sounds.

## Introduction

Recognition of complex sounds and discrimination between variations in their acoustic components is vital for social interactions in a highly social and vocal species. Little is known, however, about the acoustic basis of recognition of complex sounds. Spectrographic analyses reveal that frequency modulations (FMs) are ubiquitous in the communication sounds produced by most avian [1]–[4] and mammalian species [5]–[8]. In only a few studies, however, have scientists used the acoustic features in social calls or sequences of notes to establish their role in perceptual learning and memory [9]–[12]

Behavioral and/or neurophysiological studies on the acoustic basis of recognition of species-specific calls involve time and labor-intensive acquisition of a sometimes-large set of species-specific sounds. An analysis of their acoustic organization reveals the presence of both spectral and temporal acoustic features [6]. Some acoustic features, such as the pitch of a sound, can convey information about the mood, size and/or identity (including sex and social status) of the emitter [13]–[15]. Other features, in particular FM direction, rate (or “slope”), bandwidth (or “depth”) and duration of an FM embedded within a call likely convey meaningful information per Morton's motivation-structure hypothesis [1], [2]. Accordingly, Mongolian gerbils (*Meriones unguiculatus*) can discriminate between different directions of FM sweeps [16] and rats (*Rattus norvegicus*) can categorize FM sweeps based on either the direction or rate of modulation [17].

Speech sounds too contain frequency modulations in the form of ‘formant transitions’ where the energy rapidly and smoothly shifts from one formant (predominant harmonic) to another [1], [18]–[21]. Combinations of formant transitions (making up the sound of consonants) along with constant frequency (CF) sounds (vowels) constitute a ‘phoneme’, the acoustic-perceptual, albeit not necessarily meaningful, unit in speech sounds [22]. Similarly, for audiovocal communication in animals, a linear FM sweep by itself may not necessarily be socially meaningful but still constitute an important acoustic and perceptual unit within a communication sound. In this capacity, a multi-parametric linear FM sweep can function simply as an information-bearing element [23], [24]. Alternatively, linear and logarithmic FMs within animal sounds, similar to those in nonverbal utterances and musical sounds made by humans, may directly impact the affective or physiological state of the receiver [25]. These reasons motivated us to test rapid learning and perception of FMs in an animal model.

**Figure 1:**
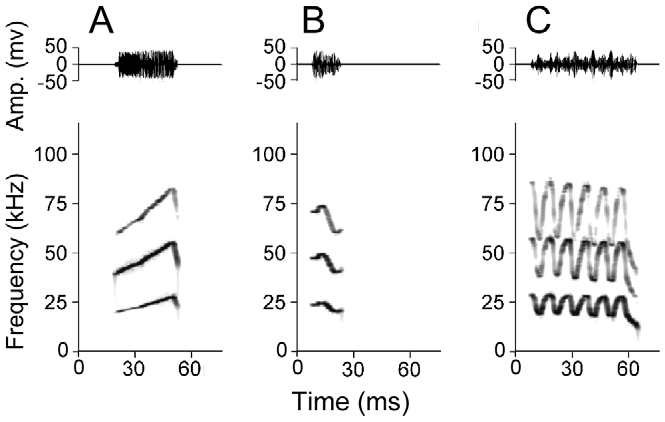
Three simple syllabic calls of *P. p. rubigenosus* containing different patterns of FMs. Amplitude envelops (above) and spectrograms (below) for (A) bent upward FM, (B) single humped FM and (C) descending rippled FM.

Mustached bats use FMs in at least two distinct ways. They use the terminal FMs in their echolocation pulses and returning echoes to compute the distance from a surface or prey (insects) when foraging in twilight [26]. They also engage in vocal interactions that accompany their daily activities in complete darkness [6]. Their social calls are spectrally and temporally complex and consist of a variety of FMs differing in bandwidth and modulation rate in the upward and/or downward directions (a few examples are shown in figure 1A–C). The meaning of these calls is also well established based on studies in a semi-natural environment [27].

We tested FM perception in mustached bats using heart rate (HR) as a quantitative denominator of the level of fear-induced autonomic activity. We demonstrate that adult bats can discriminate multiple FM parameters, such as FM direction and rate (co-varying with bandwidth) at a relatively fine-scale. For these fear-conditioning experiments, we used FMs instead of pure tones because the latter are overly simplistic from the perspective of an animal's natural environment. In contrast, FMs represent much of the spectral variability found in species-specific calls in bats as well as in many other mammalian and avian species.

Recent literature on the neural mechanisms for learning and memory suggests that a specific brain region, the basolateral amygdala (BLA), including the lateral nucleus, plays a key role in creating an association between the unconditional (aversive) and the conditional stimulus [28], [29]. To explore the neural correlates of the fear-conditioned response to complex sounds in mustached bats, we recorded local field potential (LFP) activity from the BLA prior to and post conditioning. We observed a rapid enhancement of LFPs occurring within the same time frame as an increase in HR, specifically in response to FMs paired with the aversive stimulus. This enhancement is likely part of a mechanism that is particularly active during development and allows an individual to learn the social significance of a complex vocalization produced by conspecifics. Our neurophysiological data show that specific FM parameters are able to produce response enhancement within our fear-conditioning paradigm and these may play a role in making a novel sound meaningful.

## Materials and Methods

### Ethics Statement

Bio-safety level II procedures were followed for all animal-handling and experimental protocols per guidelines established by the Centers for Disease Control. All husbandry and experimental procedures were approved by the Georgetown University Institutional Animal Care and Use Committee.

### Animal Acquisition, Maintenance and Preparation

A total of 25 mustached bats from Trinidad (*P. p. rubigenosus*) were housed in the animal care facility at Georgetown University. The mean body weight of the animals was approximately 20 grams. Bats were housed under diurnal lighting conditions (light was on from 09:00 to 14:00 hours) and supplied with vitamin-supplemented water and mealworms, *Tenebrio molitor*, (enriched with vitamin and mineral supplements) *ad libitum*. Environmental temperature was maintained at approximately 27°C and relative humidity at near 60%.

All experiments were carried out in a sound-attenuating chamber (Industrial Acoustics) that was darkened to <10 lx. Animals (6 females and 10 males) were enclosed in a soft Styrofoam body mold allowing free movements of the head and legs, which protruded from the mold. Care was taken to ensure that all animals were relaxed and complacent in the experimental environment prior to onset of experiments. During the experiment, they were continuously monitored with a low-light sensitive video camera (model BP334, Panasonic, Inc.).

### Stimulus Generation

Auditory stimuli consisted of FM sweeps synthesized to match those occurring within the 40 to 70 kHz band in species-specific social calls [6]. This range generally included rates matching the mean rate (0.67±4.65 kHz/ms in the downward direction) of FMs present within the simple syllabic call types, including frequencies that span the natural variation) in calls emitted by mustached bats (Zhang and Kanwal, unpublished data). The rate, duration and bandwidth of FM stimuli also generally corresponded to the FM tuning of neurons in the primary auditory cortex [30]. SIGNAL software (Engineering Design, Inc.) was used to generate all auditory stimuli in our study. Linear FM sweeps consisted of digitally synthesized upward and downward frequency modulations (UFMs and DFMs, respectively). The duration of direction, rate and bandwidth matched FM sweeps was 10 ms (unless indicated otherwise), which covered the duration of most FMs in calls.

FM sweeps were devoid of amplitude modulation, except for a 0.5 ms rise and fall. Stimuli were presented via a mid-line, free-field leaf-tweeter speaker (Model 423B, Panasonic, Inc.) that was placed approximately one meter away, directly in front of the animal. Sound amplitude at the animal's head was ∼75 dB SPL. The amplifier and speaker had a relatively flat (±6 dB) frequency response from 5 to 100 kHz. Further details regarding stimulus generation and presentation are described elsewhere [30].

### Fear conditioning and testing paradigms

**Figure 2:**
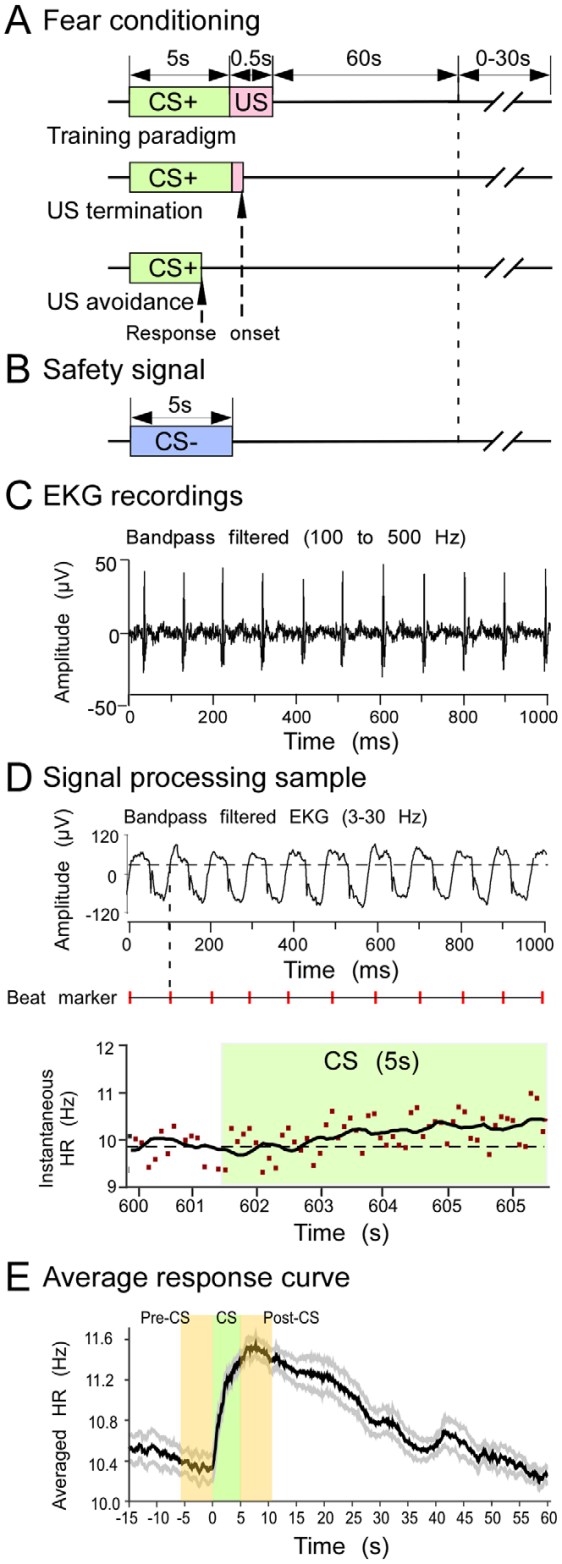
Schematic showing the CS presentation paradigm and HR recordings and analysis. (A) Illustration of the fear-conditioning paradigm. The CS+ was paired with shock to one leg. Either vocalization or leg movement terminated the US (see text for details). (B) The safety signal (CS−) was not paired with shock. (C) Band passed EKG trace showing HR recordings. (D) The filtered and amplified signal (top) used to generate beat markers (middle) that correspond (vertical dashed line) to the time of threshold crossing (horizontal dashed line). Bottom panel shows time of beat markers converted to instantaneous HR before and during presentation of CS (shaded segment) in a single trial. Note the consistently variable, but gradual increase in HR. (E) Averaged response curve obtained after CS presentations. HR increased dramatically after CS onset, and steadily declined over the next 40 s.

Two types of conditional stimuli (CS) were used in our study. The CS paired with an aversive unconditional stimulus (US) was designated as CS+ and the unpaired stimulus as CS− (Fig. 2A–B). Each CS consisted of a sequence of 100 repetitions of a tone or FM (repeating at 20 Hz for 5 s). Termination of the CS+ was followed immediately by US onset, or in relatively rare cases where the animal made an avoidance response (both discussed below). The US was a sequence of 5 monophasic current pulses (300±100 µA, 25 ms in duration, 10 Hz repetition rate) delivered using a constant-current stimulator (WPI Model A365) through two parallel silver wires placed inside a Velcro leg cuff. Currents were adjusted across trials to prevent adaptation to the US. Inter-stimulus intervals (ISIs) were randomized (ranging from 60 to 90 s) to prevent interval conditioning (Fig. 2A–B).

Animals were exposed to 25–50 presentations of CS+ and CS− on each training day. All CS+ presentations were reinforced with leg shock during this phase of training. To avoid the possibility that mustached bats may have a preexisting (i.e. unconditioned) response to a conditional stimulus (CS+), in a few cases, stimuli with different FM parameters were chosen to be the CS+. If after 3 to 4 days of training, the animal did not show any consistent conditioned response, it was eliminated from the experimental set. Those that showed a significant change in HR (P<0.05) underwent discrimination training.

For discrimination training, FM stimuli chosen as CS− differed from the CS+ in either direction, or a combination of duration/rate or bandwidth/rate. A DFM (40 to 30 kHz sweep in 10 ms) paired with shock was used as CS+. The CS+ and CS− were randomly intermixed in the same block of trials and presented 25 times each. After an animal was trained with one of the three pairs above, changes in HR evoked by CS+ versus CS− presentations were determined (detailed descriptions of HR calculation and statistical analyses are provided below). If the bat showed a nonspecific HR increase following both CS+ and CS− presentations, and the response to the CS+ and CS− were statistically indistinguishable (independent-samples t-test, n = 25, P>0.05), then the CS− was presented 100 or more times (for extinction of the CS− response) until the bat showed discrimination between the CS+ and CS−. Animals were trained to discriminate between each of the three pairs of stimuli across separate days.

Three bats were trained to discriminate between an additional CS+/CS− pair differing in rate (FM versus pure tone at 60 kHz) and bandwidth, and were subsequently tested on 9 additional stimuli of intermediate rates and bandwidths. FM rate of the 10 DFMs ranged from 0.1 to 1.0 kHz/ms (increasing in steps of 0.1 kHz/ms) and in bandwidth, centered at 60 kHz, from 1 to 10 kHz. The durations of the tone and all DFMs were fixed at 10 ms. For initial discrimination training, the DFM with the largest rate and bandwidth (10 kHz sweeping from 65 to 55 kHz) was used as CS+, and the pure tone was used as the CS−. Once the animal showed discrimination between this stimulus pair (independent-samples t-test, P<0.05, see below), HR was measured in response to all of the 12 stimuli. The stimulus set used for testing comprised of 40 shock-paired presentations of the CS+ (to prevent extinction), 8 unpaired presentations of the CS+ (i.e. extinction testing trials), and 8 presentations of each of the 10 CS− stimuli (9 novel DFMs and the original pure tone). Stimulus order and ISI were randomized as described above.

### Vocalization and leg movements

All animals were given the option to avoid shock altogether by either vocalizing or leg flexion during presentation of CS+. This aspect of the experimental design was motivated by previous literature [31], [32], which suggests that the direction (increase versus decrease) of HR responses (see below) to fear conditioning can depend on whether the animal perceives that it has some control on the outcome of CS+ during training. Any vocalizations emitted by the animal, which crossed a set threshold, were acquired by a high frequency microphone (model 9569, ACO Pacific Inc. USA) and filtered between 1 and 100 kHz. Vocalizations were detected by threshold crossing of the recorded audio signal. The threshold was set to lie above the maximum level of recorded CS playback. Threshold crossings were re-sampled to have a maximum rate of 100 Hz to provide event markers for quantifying vocal utterances.

Leg flexion acted as a marker of somatic motor response. The Velcro leg cuff was attached with a light string to a narrow plastic strip patterned with a high-contrast grating, which was passed between an IR emitter and detector on a circuit board. Leg movement translated the grating through the beam, leading to transitions from opaque to translucent portions of the grating. Each opaque bar broke the beam and the translucent spacing in-between allowed it to pass. Each transition triggered the transmission of a 3 ms data packet, resulting in a record indicating the occurrence of leg movement with a sampling resolution of 333 Hz.

### Data acquisition

The electrocardiogram (EKG) was recorded differentially from two silver wire leads firmly pressed against the dorsal surface of each thumb region. We found that a relatively low frequency, narrowband EKG (band-passed 3–30 Hz) was less prone to noise contamination and was a convenient signal from which to generate R-R interval markers (described in detail below). Band-passed EKG (trace shown in figure 2C) was amplified 10,000 times and recorded at a sampling rate of 5 kHz with the Power1401 hardware and Spike2 software (Cambridge Electronic Design). EKG was recorded continuously until the end of each trial in our experiments, including during instrumental avoidance learning.

LFP activity was acquired with tungsten microelectrodes from head-restrained animals before and after applying the fear conditioning procedure. Electrodes had a tip diameter of ∼10 µm and an impedance of 0.5 to 1.5 MΩ. Signals were amplified and filtered between 1 and 300 Hz and recorded digitally at a sampling rate of 5 kHz using Spike 2 software. The electrode was slowly advanced into the brain using a custom-made stereotaxic system [33] and predetermined co-ordinates for the BLA and relative recording positions were noted. Stereotaxic co-ordinates were recorded and electrode position was marked with an electrolytic lesion and verified using standard histological procedures.

The search stimuli consisted of 14 FMs at different rates (increasing in steps of 0.2 kHz/ms) matched in duration (10 ms) and in center frequency (60 kHz). FM bandwidth (from 0 kHz to 14 kHz) co-varied with rate; a single FM stimulus was played every 500 ms; the first FM in the sequence was a DFM sweeping from 65 kHz to 55 kHz. The whole sequence was repeated 100 times and LFPs were averaged to analyze response parameters. An FM eliciting a moderate response (relative to the peak response obtained for the best FM) was used as the CS−. Likewise, a sub-optimal FM was used as the CS+, to avoid a ceiling effect from preventing response enhancement after repeated pairing with the US. Responses to the CS+ and CS− were obtained from the same recording site before and after conditioning. The fear conditioning procedure was similar to that used to evoke HR changes to the conditioned response, except that the trial number for CS+ and CS− presentations was limited to between 30 and 100 per CS.

### Processing and analysis of data

Spike2 software was used to analyze the EKG data. The EKG recordings were high-pass filtered to remove any DC shifts due to animal movement, and smoothed (250 ms sliding window) for generating reliable timestamps for each cardiac cycle. Time stamps (beat markers) were extracted from positive-going threshold crossings (Fig. 2D). Instantaneous HR was calculated as the reciprocal of each interval between consecutive timestamps (i.e. each pair of beat markers). Upper and lower bounds for instantaneous HR were used to identify intervals with missing or erroneous markers. Linear interpolation was used to fill in missing beat markers when animal movements degraded signal quality. Animal movement and marker interpolation rarely corresponded to the stimulus playback period. The instantaneous HR was averaged for all CS+ versus CS− presentations and the averaged response curves were smoothed with a 0.5 s sliding window (Fig. 2E).

The averaged HR response curve to a CS typically reached peak amplitude within 3–7 s of the onset of the CS sequence before gradually returning to baseline. The location of peak responses was confirmed visually to be relevant (i.e., occur within 20 s following CS onset) before proceeding with calculation of response magnitudes using custom-written scripts. Response magnitudes were calculated for a 6-second window centered on the peak of average waveform (3 s on both sides of the peak value) following stimulus onset, and the average HR during the 6 s immediately preceding the presentation of the stimulus. Change in HR (∂HR) was calculated as the difference between the HR in the pre- and post-stimulus time windows; HR change was also expressed as a percentage of the mean pre-stimulus HR.

Responses of male and female bats were not significantly different (independent-samples t-test, P>0.05), and were pooled for further statistical analysis. Means are given with their standard deviation unless stated otherwise. For checking the regression between the sensitivity of HR to FM rates, ∂HR to each rate was normalized by dividing the HR change evoked by CS+ without shock (the target FM). Means are given with their standard deviation (unless stated otherwise). Statistical analysis was conducted using commercial statistical software SPSS 17.0 (SYSTAT, Inc.) and a two-tailed t-test (at the 0.05 level unless stated otherwise) was used to determine significance. Parametric tests were applied to data that were normally distributed (one-sample Kolmogorov-Smirnov test, P>0.05); otherwise nonparametric tests were used. Pre- and post-stimulus HR values were compared using the paired-samples t-test. The HR responses evoked by the CS+ and CS− were compared using the independent-samples t-test. Repeated-measures ANOVA was used to analyze HR responses to different FM rates in three bats.

## Results

### Instrumental Avoidance Learning

**Figure 3:**
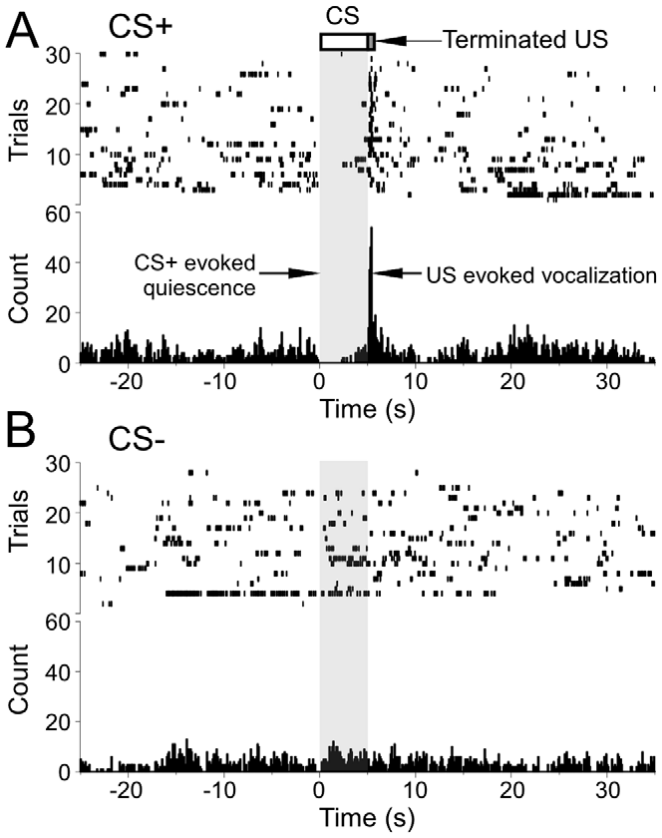
Fear-induced learning employing vocalization as the operant conditioning paradigm. (A–B) Raster plots for event markers and PSTHs of vocalization markers to show selective suppression of spontaneous echolocation by CS+ presentations. (A) Echolocation pulses are suppressed during playback of the CS+. The bat vocalized with short latency after delivery of a shock pulse at the end of the CS+. Unfilled rectangle and vertical grey bar indicates timing and duration of the stimulus. (B) The CS− did not influence the rate of echolocation.

Preliminary tests were carried out in several animals to determine whether mustached bats show FM discrimination in our stimulation and recording setup by making reliable vocalizations as avoidance responses to the CS+. Vocal responses elicited by the CS+ were uncommon, though one animal did learn to avoid the CS+ based on a vocal response. This animal vocalized on 88% of CS+ presentations with shock, and on 76% of CS+ presentations without shock (testing), as compared to 43% of CS− presentations. Conversely, another animal that had undergone several fear conditioning sessions emitted echolocation pulses spontaneously and throughout one of the sessions. In this animal, the CS+ (DFM ranging from 55 to 50 kHz with a 90 ms duration) elicited vocal freezing (suppression of ongoing vocalizations; Fig. 3A). The CS− of the same duration and bandwidth, but different (UFM) direction and frequency range (30 to 35 kHz), failed to elicit a freezing response (Fig. 3B). There was no difference in HR for the ‘vocalization’ and ‘no-vocalization’ conditions during testing with CS+ (nonparametric Chi-square test, P = 0.13).

**Figure 4:**
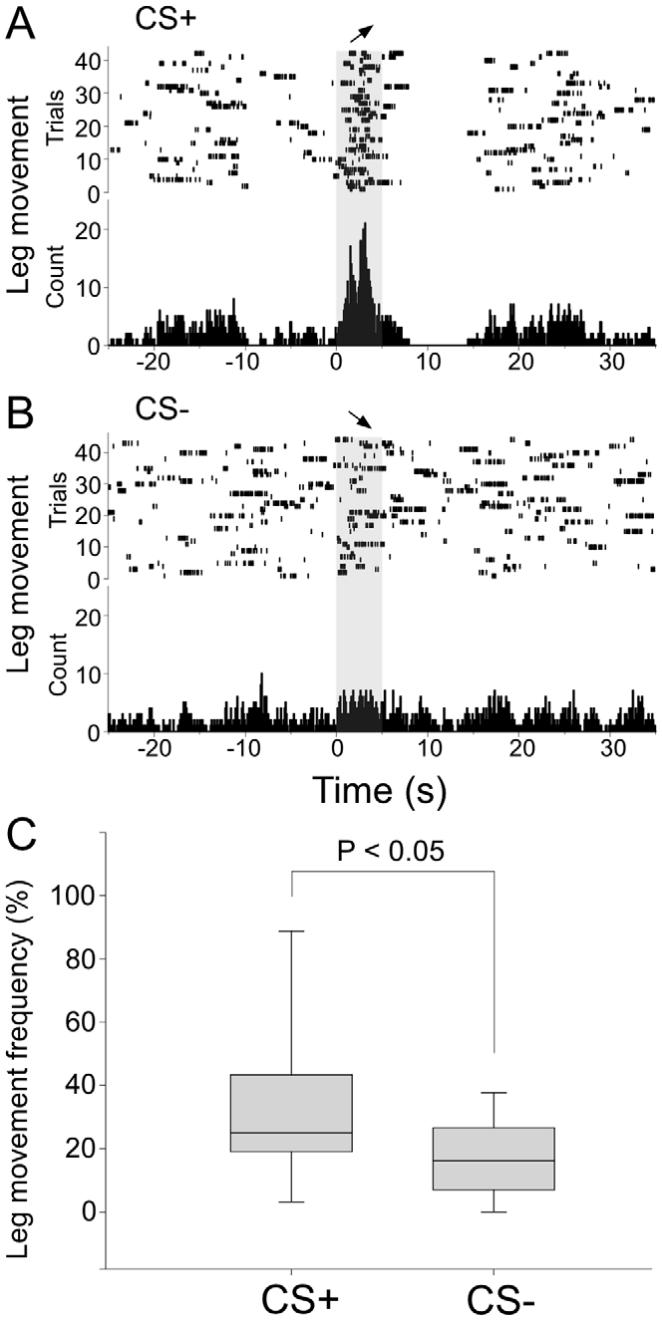
Fear-induced learning employing leg movement as the operant conditioning paradigm. (A) Raster plots and PSTHs of leg movement transiently increased during the CS+ (UFM) and decreased following CS+ offset, whereas (B) the CS− (DFM) did not influence the frequency of leg movement. FM direction is indicated by arrow. Box plots of leg flexion events analyzed in 5 animals (C) showing a significant difference in leg flexion between the CS+ and CS−. Solid lines in boxes are medians, boxes surround 50% data and whiskers are 5th and 95th percentiles.

Leg movements were used as another avoidance responses to CS presentations. Figure 4 shows raster plots of leg flexion event markers and peristimulus time histogram (PSTH) during presentation of CS+ (a 40–70 kHz UFM; Fig. 4A) and CS− (a 30 kHz tone; Fig. 4B). This animal avoided shock for 67% of the CS+ presentations (n = 44). Five animals showed significantly more leg flexion during CS+ presentations (43.5%, n = 131) than during CS− presentations (27.6%, n = 196; Mann-Whitney U test, U = 7.00, P = 0.025). These experiments demonstrated the feasibility of testing FM discrimination for direction and other parameters at the perceptual level within our stimulation and recording setup. No difference in HR was present in the two conditions: CS+ with leg withdrawal and CS+ to which there was no leg withdrawal in five bats (Mann-Whitney U test; P = 0.15).

### Basic properties of HR responses

HR provided a robust measure for discrimination between FM sweeps. The average resting HR of all animals placed in the holder was 9.7±0.86 Hz or 578±29 beats per minute (BPM). Males had slightly higher resting HR of 591±44 BPM compared to females (578±32 BPM). HR gradually increased from baseline at the onset of CS presentation. HR reached its peak value within a few seconds of stimulus offset. On average, the change in HR to the CS+ without shock was about 16 BPM.

### HR as a measure of fear conditioned learning

**Figure 5:**
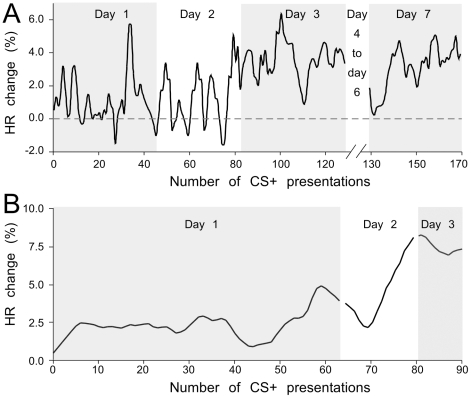
Two examples of learning curves using HR recordings. Lines are smoothed (0.5 s and 1.0 s sliding window in A and B, respectively). Line plot of HR is expressed as percentage change above average pre-stimulus values representing the “learning curve” for first three training sessions in two bats. Each bat acquired a robust and relatively stable response on the third day that was rapidly re-established on day 7 in the first bat (A).

Most bats developed a conditioned response within the first three training sessions. Figure 5 shows two examples from 10 of the16 bats tested that developed increases in HR to the CS+ over the course of 15 to 30 pairings with the US. Responsive animals showed HR fluctuations with overall increases of 0.2–5.9% relative to pre-stimulus values on the first training day (Fig. 5A). Both males and females exhibited acquisition of the conditioned response. However, a relatively high percentage of males learned to discriminate between the CS+ and CS−. Male bats also tended to learn more quickly than females, but the learning curve was not significantly different between males and females (Mann-Whitney U test, U = 25.50, P = 0.624). During retraining, the slope of the learning curve was much steeper than *de-novo* training as reflected in ∂HR (Fig. 5B). In the second example, the animal learned to near-criterion level in the first 5 trials and then showed a plateau with a decline over the next 50 trials before rapidly picking up to a 5% change in the next 10 trials. The HR increased significantly after the CS playback on the very first trial, prior to retraining, on day 2. Most bats showed quick recall and response enhancement on the third day after presentation of 20–40 trials of reconditioning.

### Discrimination of FM direction, duration and bandwidth

Animals were trained to discriminate FM sweeps in the opposite direction (6 bats), with different bandwidths (11 bats), and different durations (2 bats). Modifying the primary variable (either duration or bandwidth) between CS+ and CS−, while holding the alternate parameter constant also produced changes in FM rate (the covariate). A primary variable was one that was the main target of modification within a specified range without regard to changes in the covariate.

**Figure 6:**
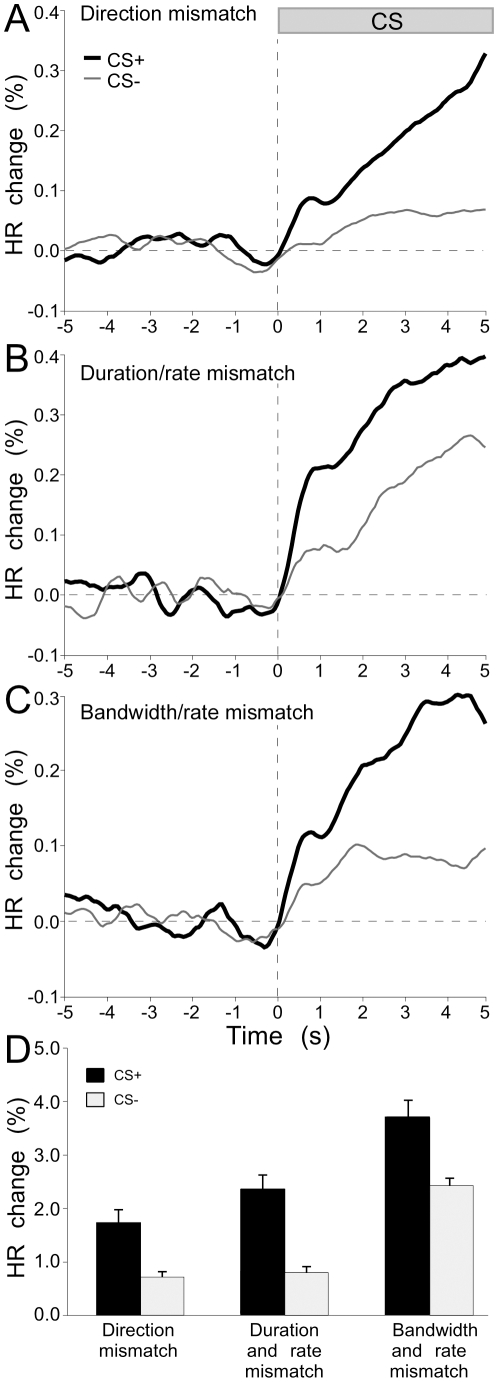
Cumulative line plots for representative examples of peri-stimulus HRs in response to conditioning with three different FMs. CS+ was a steep DFM (40 to 30 kHz sweep in 10 ms; rate 1.0 kHz/ms) FMs used as CS− differed (A) in direction (UFM), (B) in duration (100 ms) and rate (0.1 kHz/ms), and (C) in bandwidth (60 kHz to 10 kHz) and rate (0.5 kHz/ms). Line plots indicate instantaneous HR to the presentation of FM stimuli. Each black trace represents the HR in response to CS+, and each grey trace shows HR on trials with one of the three CS− stimuli. CS onset occurs at time zero (vertical dashed line). (D) Bar graph of data to show ∂HR for CS+ (black bar) versus CS− (grey bar) for each of the same three stimulus conditions. Error bars represent standard error of the mean.

Data shown in figure 6A–C were obtained from three animals and show significantly different HR values for FMs differing along the three parametric dimensions (ignoring rate as a covariate). In each case, figures 6A–C, traces of averaged instantaneous HR show a smaller relative amplitude and slope of the HR increase following the CS− than CS+. This indicates the graded nature of the HR response indicating both the perceptual difference between the CS+ and CS− as well as the perceptual similarity of FMs, being of the same stimulus modality.

Figure 6D shows the means of discrimination performance in 5 animals for the test parameter indicated on the X-axis. Mean HR was measured during the CS period and averaged across CS presentations (25 – 200 per stimulus type). Mean ∂HR values were significantly different for each pair of stimuli tested (one-tailed, paired-samples t-test, P<0.01).

### Sensitivity of HR to FM rate

**Figure 7:**
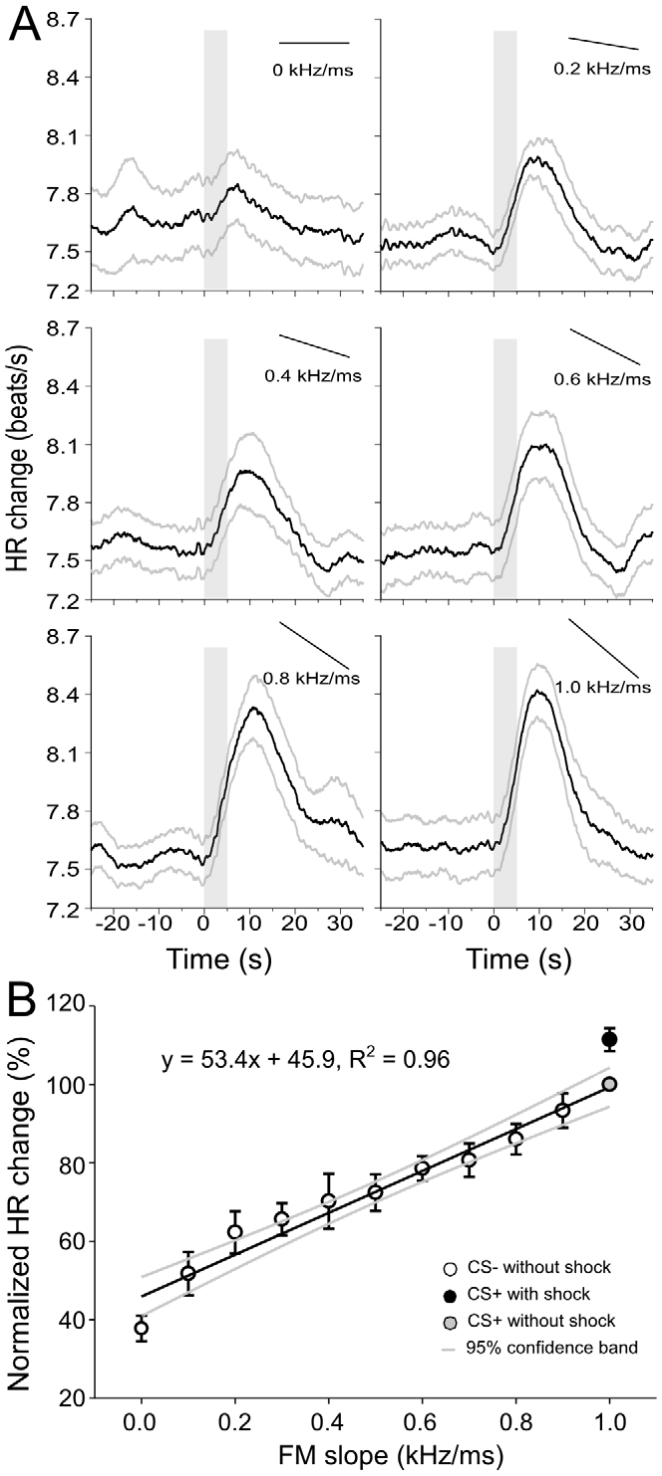
Dynamic correlation between FM rate and HR responses. (A) The traces represent the average instantaneous HR of one bat to 10 presentations of a CS− and CS+ without shock. The vertical grey bars mark the presentation period of the stimulus. The solid black bars at the upper-right corner in each panel denote the rate of the FM. (B) Regression plot (averaged from three bats) for FM rates ranging from a pure tone (0 kHz/ms) to 1.0 kHz/ms (step size of 0.1 kHz/ms). HR obtained to CS+ with shock during training (filled black circle) was not included when fitting the regression line. The data were best fit by a straight line suggesting that HR increased linearly with increasing rates approaching CS+. No significant difference was found in the HR evoked by CS+ with shock (filled black circle) and the CS+ without shock (target FM, filled grey circle) independent samples t-test, P = 0.992).

Since FM rate was a confound (covariate) in discrimination tasks for bandwidth and duration, the effect of rate (covaried with bandwidth) as the primary variable on HR was separately tested. FMs were tested in 3 animals. All test FMs with rates corresponding to CS− evoked tachycardia (HR increase is indicated as ∂HR) ranging from 0.2% to 7.6%. During and after training, ∂HR to CS+ ranged from 3.8–6.2% (n = 3 bats, 208 pairings) above baseline. In each animal, random presentation of an array of FMs sweeping at different rates resulted in a progressive increase of HR for a rate of 0 kHz/ms (pure tone at 60 kHz) to 1.0 kHz/ms (target FM) (Fig. 7A). To quantify the rate of change of HR in each animal, the slope of the best-fit line was calculated for each bat. In each case, a straight line fit the data points better than any other function. An average regression coefficient for data combining the normalized ∂HR for all three bats was 0.8515 (Fig. 7B) (F = 110.77, df = 1, P<0.0001). Repeated measures ANOVA was used to test for a main effect of FM rate on change in HR. The analysis revealed that the HR was significantly different between the different treatments (the array of FM rates presented to each animal after conditioning). The F-values for a df = 10 obtained for each animal were as follows: (bat 17, F = 17.01; bat 22: F = 5.47; bat 24: F = 3.86; P<0.001). There was no significant difference between the 10 FMs at different rates (0.1 to 1.0 kHz/ms) corresponding to different CS− presented prior to conditioning (repeated measures ANOVA; F = 1.01, df = 10, P = 0.44). Rates faster than the CS+ (2.0 and 4.0 kHz/ms) and well within the range of FM rates present in natural calls were also tested in three animals. The result showed that the two CS− with rates faster than CS+ also resulted in a smaller ∂HR to CS− (0.39±0.30% for 2.0 kHz/ms: n = 166; and 0.34±0.29% for 4.0 kHz/ms: n = 166) than that (1.27±0.29%, n = 136) to CS+ (rate of 1.0 kHz/ms) without shock (independent-samples t-test, t = 2.066, df = 300, P = 0.04; t = 2.241, df = 300, P = 0.026, respectively). This demonstrated that HR does not increase monotonically with FM rate.

### Plasticity to FM direction in the amygdala

**Figure 8:**
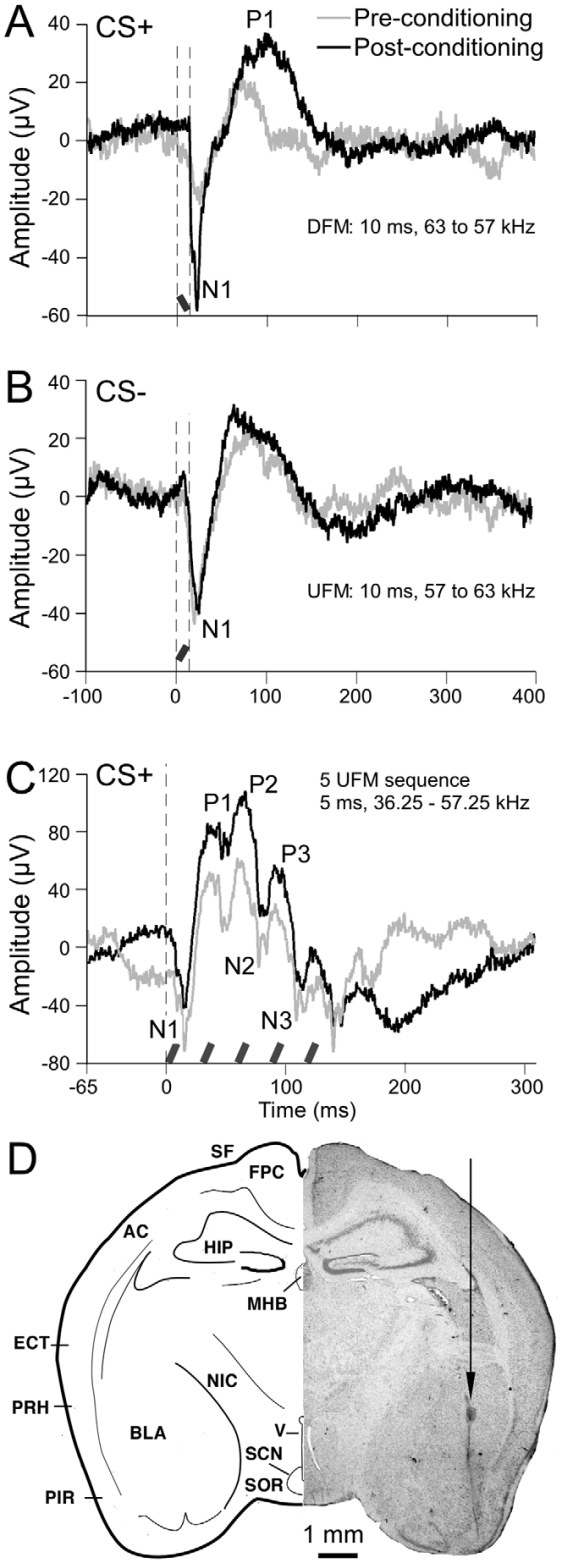
Two examples of US induced enhancement of the LFP to CS+. Line plots show averaged LFPs evoked by FM sweeps before (grey trace) and after (black trace) conditioning. (A) LFP responses after 100 repetitions of the target stimulus CS+ (a single DFM at a rate of 0.6 kHz/ms). (B) LFP response to CS−, and (C) LFP response to a sequence of 5 FM sweeps (CS+) pre- and post-conditioning presented at rate of 40 Hz; onset is indicated by vertical dashed line. Responses were obtained from depths of 3.71 mm (A and B) and 3.50 mm (C) from the brain surface and ∼3.1 mm from the midline. Vertical dashed lines and black/grey diagonal bars indicate stimulus onset and offset. Labels indicate enhanced peaks P1 to P3. D. Charting (left) and photomicrograph (right) of a histological section in the transverse plane to show the location of the electrolytic lesion (arrow) at a LFP recording site. AC: auditory cortex, BLA: baslolateral amygdala, ECT: ectorhinal cortex, FPC: frontoparietal cortex; MGB: medial geniculate body; MHB: medial habenular nucleus, NIC: nucleus of the internal capsule, PIR: piriform cortex, PRH: perirhinal cortex, RS: rhinal sulcus. SCN: suprachiasmatic nucleus, SF: Sylvian fossa, SOR: retrochiasmatic part of the supraoptic nucleus, v; fourth ventricle.

CS-US pairings during conditioning showed enhancement of both negative and positive peaks in the LFPs elicited by the CS+ post-conditioning. The CS− consisted of the same FM sweep (100 ms at 0.6 kHz/ms) played backwards (opposite direction). Figure 8 A–C shows two examples of such an enhancement at two different locations in two animals. Response magnitude to the non-target stimulus (CS−) a single UFM was relatively unchanged post-conditioning with the CS+. The first negative peak (N1) has a latency of 20 to 30 ms and likely corresponds to spiking in cells near the tip of the recording electrode. Total response duration was on the order of 170 ms and did not change specifically for CS+ post-conditioning.

There was an increase in onset response latency of 4 ms for CS+ and a decrease in latency of N1 from response onset by ∼7 ms accompanied by a doubling of the peak-to-peak amplitude (compare figures 8A–B). At a second location, the CS+ consisted of 30 CS-US pairings of a sequence of 5 FM sweeps repeated at a rate of 40 Hz. A significant response enhancement (∼2.5 times increase in response magnitude) of N1 was observed with subsequent positive peaks also showing enhancements in response to the first 3 FMs in the sequence within the CS+ (pre and post-conditioning) (Fig. 8C). Multiple peaks were not present in the LFP response to CS+ and CS− at the first recording location (Fig. 8A–B) since only one FM was presented. The negative phase that follows is an additional temporal feature that may result from spike suppression. This was not observed in response to the presentation of a single FM. Not all locations showed an enhancement and the specificity of the response deteriorated with supra-optimal stimulation with the US. A small suppression of the positive peak in the LFP was also observed at a few recording locations. Figure 8D shows the location of the recording site (for data in figures 8A and 8B) marked by an electrolytic lesion.

## Discussion

### Behavioral responses to FMs in bats and other species

For social communication, the ability to recognize and discriminate between different call types and call variants is a key requirement. The detection of FM parameters may be a significant component of this ability. In fact, in the distress calls of mustached bats (a long wrinkled FM call) and starlings (*Sturnus aulgaris*), a “secondary modulation” is sometimes superimposed on a “primary modulation” of the carrier frequency [6], [34] suggesting that frequency modulations enrich the variety and complexity of calls. We also know from Morton's motivation-structure hypothesis that different types of sound patterns, including FMs have a universal significance in avian and mammalian species [1].

Such discriminatory capabilities could also be meaningful during echolocation, given that a small modulation of the CF can occur within the Doppler-shifted echo returning from targets during the search phase of echolocation behavior in this species. Calculations show that upward FMs with slow rates (<0.01 kHz/ms) can be generated for the second harmonic of the Doppler-shifted echo CF if the bat accelerates and turns directly towards a target (Mueller, R., personal communication). FM rates corresponding to those tested here, however, are impossible to attain during normal flight. For all other off-center targets, the highest probabilities are for encountering small DFMs with rates ∼0.01 kHz/ms. They did not, however, approach the FM rates presented in our experiments that were one to two orders of magnitude larger. The stereotypic DFM in the second harmonic in the echolocation pulse has a fast modulation rate (4 kHz/ms). This FM rate is at the high end of what was tested in the experiments reported here. Therefore, discriminatory capabilities shown in our study are less meaningful from the perspective of echolocation.

By virtue of the nature of FMs, one or more acoustic parameters co-vary with different FM test parameters investigated in this study. To completely rule out the role of rate in FM discrimination, it is necessary to test FMs differing in bandwidth (matched in rate, center frequency, upward or downward direction) and duration. This was not attempted in the present study. Other parameters, e.g. rate and starting and ending frequencies (in the case of stimuli with shifts in center frequency) could also be important making it difficult to specify which parameter is the most relevant for triggering changes in HR. One would need a large number of controls and parameter combinations to test the contribution of every possible parameter and this may change with the combination tested. Controlling for the more important FM parameters, data from the three conditions shown in figure 6 and the FM rate modification in figure 7 collectively provide evidence for a relatively high acuity in mustached bats for FM discrimination along multiple acoustic dimensions.

Most behavioral studies of FMs use logarithmic FMs also referred to as “fast FMs” that cover a frequency range greater than 10% of the central frequency on a relatively short time scale [16]. One major rationale for the use of logarithmic FMs is that they more closely conform to cochleotopic organization and thus, unlike linear FMs, ensure equivalent acoustic stimulation across audible frequencies. In a recent study, Gaese et al., used a two-alternative-forced-choice-paradigm in rats to show that discrimination of upward versus downward direction in logarithmic FM-sweeps was reduced with increasing sweep speed between 20 and 1,000 octaves/s [16]. They found that discrimination performance declined with increasing lower frequency boundary of FM sweeps, showing an especially strong deterioration when the boundary was raised from 2 to 4 kHz. In comparison, FMs used in our study spanned a relatively short frequency range. Logarithmic FMs that span a wide frequency range are largely absent in mustached bat calls [6]. Hence, taking a neuroethological approach, we looked at FMs present within species-specific calls that are commonly heard by conspecifics (see also [35]. These calls contain relatively short bandwidth FMs (sometimes as part of a complex FM pattern) within any one harmonic. Furthermore, a set of linear FMs can simulate continuous rates of frequency change, such as the descending humped FM. Therefore, similar to orientation tuning in the visual cortex [36], [37], it is conceivable that perception of more complex patterns of FMs in fact emerge from a tuning at the neural level to narrowband and/or linear FMs.

Whether FMs should be considered simply as a sequence of brief pure tone bursts successively stepped up or down, or as more holistic acoustic features remains a point of contention among researchers [38]–[41]. Species, such as mustached bats, that use a rich repertoire of calls specialize in the detection of a FM sweep as a modulation continuum rather than as a sequence of pure tone steps [42]. Other species that do not use sounds for a specialized function may still be trained to discriminate FMs with high acuity. In still others, FM parameters may not be sharply resolved at the perceptual level, especially if they are not ethologically relevant. In this case, FM responses may depend on a “trigger frequency” present within the FM [43]. Despite numerous physiological studies [44]–[47], there are relatively few behavioral data on the perception of FMs in nonbat animal models [20], [35], [48]. Neuronal as well as behavioral experiments describe FM detection and discrimination in the Mongolian gerbil [48], [49] and categorical perception of FM direction and rate in the rat [16], [17], [50]. Mercado et al. [17] observed that rats do not perform as well for categorization of FM range as they do for FM direction and rate. They concluded that rats likely would be better able to categorize FM sounds that span a narrower range of nonoverlapping frequencies because their neural representation would be spatially separable at multiple levels within the auditory system.

### FM Discrimination: neurobiological underpinnings

Rate, bandwidth, central frequency, and modulation direction are four key parameters dictating neural responses to FMs in the primary auditory cortex of mustached bats [30], [51]. In the study on mustached bats [30], responses of cortical neurons to FMs ranged in bandwidth between 0.44–7.88 kHz, on average and responded more to FMs with the larger bandwidths than those with the least. They also respond well to rates ranging from 0.04 to 4.0 kHz with a preference for slower rates [30]. In rats, bell-shaped tuning curves were obtained for responses of neurons in the rat inferior colliculus to the rate, bandwidth, and amplitude of linear FMs [52]. These types of curves are indicative of tuning to a particular FM parameter. Neural tuning to an FM parameter supports the idea that the auditory system extracts information about specific FMs, which could then be used for discriminating one call type from another. The FM response curves of single neurons in this and other species indicate that the neurons do not respond in an all-or-none fashion to particular FM parameters, such as rate. The diminished, but distinct, response of neurons to FMs with parameters approaching that of the tuned FM may lead to a level of perceptual ambiguity expressed in the graded, but quantifiable HR response to CS− in our study (see figures 6 and 7).

Studies on the neural representations of FMs in the auditory cortex have focused on a variety of different FM classes, including linear [43], [53], [54], logarithmic [44], [55] and sinusoidal FMs [38], [56]. Studies using logarithmic FMs in the ferret demonstrated an overall preference for upward FMs [43], [57]. Neurons responding to linear FMs also tend to show a greater preference for a particular direction of modulation than those to logarithmic FMs. Reversing a communication call, such as the bent upward FM, reduces the peak response magnitude in the more specialized, but not all cortical neurons [23], [58]. In the pallid bat (*Antrozous pallidus*), neurons in the primary auditory cortex have a downward directional preference and bell-shaped response curves to FM rates [59].

### Fear conditioning to pure tones versus FMs

In rats, arterial pressure and behavioral responses, but not HR, was found to reflect associative conditioning [60]. In rabbits, HR deceleration was the main response to fear conditioning to the presentation of pure tones [61]. This could be a species difference or result from the use of pure tones as conditional stimuli. Our data show that mustached bats can perceive relatively small differences in the rate of a linear FM sweep in addition to discriminating upward from downward FMs and between combinations of other FM parameters. The acuity in the detection of differences in the rate of an FM are at least on the order of 0.1 kHz/ms. This translates into a change of 0.16% at the carrier frequency of ∼60 kHz and is in the same general range (∼0.1%) obtained for juveniles of the lesser spear-nosed bat, (*Phyllostomus discolor*) [35]. In the latter species, a two alternative forced-choice task yielded a difference limen for modulation frequency of 2.42 Hz for spectrotemporal resolution of sinusoidally modulated signals; we did not try to a determine difference limen for detection of any FM parameter in mustached bats.

Among the parameters tested, FM direction and rate have a perceptual advantage in that they are constant throughout the stimulus duration and this information is available both early on and at almost any time a listener attends to a stimulus. FM duration and bandwidth, on the contrary, can only be determined if a listener pays attention to at least the beginning and end of the stimulus. It is not surprising therefore that in the study on categorization of FMs, rats were better at using direction and rate information than range information [17]. FM rate may constitute a key information-bearing parameter for call discrimination. Based solely on our autonomic response study, it is difficult to know as to which parameters HR is most sensitive to either at a conscious or subconscious level for discriminating between two FMs. Given our FM response data and other behavioral and neurophysiological studies, however, it is highly likely that many animal species, especially bats, are able to detect multiple FM parameters.

### Neural structures underlying fear conditioning

Auditory fear conditioning is a useful paradigm for understanding mechanisms that link perception of environmental sound to behavioral readiness and execution. This perceptual ability may emerge either from genetically determined connectivity and properties of neurons within various brain circuits and/or from learning. Behavioral studies coupled with lesions [62]–[64], pharmacological manipulation [65]–[67], or neurophysiological recordings [68]–[71] stress the role of learning and demonstrate that the amygdala is a critical brain structure involved in fearful and appetitive responses to conditional stimuli, including their extinction and reinstatement [72]–[74].

**Figure 9:**
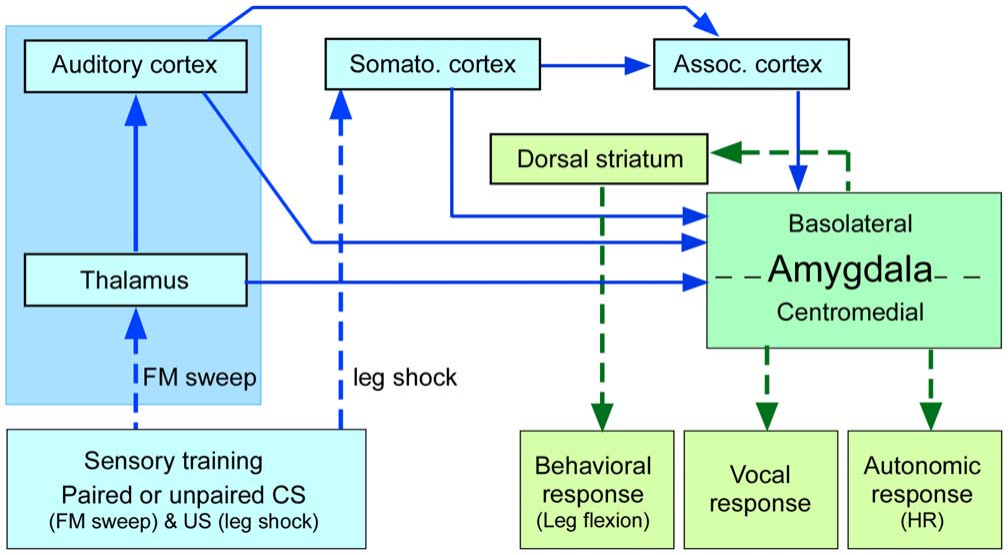
Schematic showing major brain regions involved in the generation of a fear-conditioned response. Associations between the CS and US are created in the BLA from auditory and somatosensory inputs. Leg movement is mediated vja projections of the BLA to the dorsal striatum [84], whereas HR and vocalizations are mediated by parallel pathways from the BLA to motor centers within the brainstem via the centromedial amygdala [85], [86]. BLA has been proposed as a locus for associative plasticity [87]. Solid lines indicate direct neuronal projections; dashed lines indicate indirect (multilevel) projections. Connectivity diagram does not show reciprocal connections between amygdala and auditory brain regions. Sensory processing areas and inputs are indicated in light blue, motor centers and outputs are indicated in green and sensorimotor interface (amygdala) in bluish green.

Auditory fear conditioning involves integration of parallel auditory and somatosensory inputs to the lateral nucleus of the amygdala [75], [76] and connections of the BLA with the central nucleus. The central nucleus of the amygdala in turn projects to the lateral hypothalamus and brainstem target areas that directly mediate fear and anxiety [77]. A simplified scheme illustrating the central role of the amygdale in eliciting various behavioral and autonomic responses and its connectivity with the auditory systems is shown in figure 9. In adult big brown bats, *Eptesicus fuscus*, neurophysiological studies show that combined electric stimulation of the auditory and somatosensory cortices evokes collicular and cortical plasticity and this is augmented by electric stimulation of the basal forebrain [78], [79]. Although some of the conclusions reached in these studies differ from those of behavioral studies on plasticity in the auditory cortex of rats [80], the role of the BLA, including the lateral nucleus, as the sites of this plasticity is well established [81]–[83]. Our behavioral and neurophysiological data in the same species help to expand the role of the BLA in the learning of FM sweeps, which represent the next level of acoustic complexity in comparison to pure tones.

A response enhancement within LFP activity recorded in the BLA in our study also supports the discriminability of FMs at the neuronal level. Response enhancement represents a sensitization of BLA neurons to incoming auditory inputs. It remains unclear if the response enhancement in the LFP is due to single neurons firing at higher rates or the synchronous firing of a larger number of neurons compared to the preconditioning state. Several studies have further elaborated the neural circuitry mediating conditional responses to tones [62], [63], [69] that can be considered to be applicable to FMs as well.

In summary, our study demonstrates that partially restrained, awake bats can discriminate between multiple FM parameters as indicated via fear conditioning and HR as an indicator of acoustic similarity. This relatively convenient, quantitative and noninvasive method of testing FM discrimination opens up new opportunities for studies of auditory perception and the role of complex sounds in modifying physiological (autonomic) and motivational states in a small mammal. The mustached bat is already an excellent model for studies of neural processing at all levels of the auditory system. Our fear conditioning preparation is compatible with that used for obtaining neural recordings and this paves the way to quantitatively evaluate the role of different brain regions and/or neurotransmitters in associative learning and neural encoding of FMs [47]. It also facilitates future studies of the neural mechanisms underlying neural plasticity for the perception of complex sounds as well as the acoustic basis for the communication of affect.
